# Health-related quality of life following lower-extremity constraint-induced movement therapy after stroke: an intervention study

**DOI:** 10.3389/fneur.2026.1826682

**Published:** 2026-09-09

**Authors:** Therése Brändström, Annika Sefastsson, Håkan Littbrand, Per Wester, Britt-Marie Stålnacke, Ann Sörlin, Xiaolei Hu

**Affiliations:** 1 Department of Community Medicine and Rehabilitation, Rehabilitation Medicine, Umeå University, Umeå, Sweden; 2 Liljeholmskliniken, Stockholm, Sweden; 3 Department of Community Medicine and Rehabilitation, Geriatric Medicine, Umeå University, Umeå, Sweden; 4 Department of Public Health and Clinical Medicine, Umeå University, Umeå, Sweden; 5 Department of Clinical Science, Karolinska Institute Danderyd Hospital, Stockholm, Sweden; 6 Department of Community Medicine and Rehabiliation, Umeå University, Umeå, Sweden

**Keywords:** constraint-induced movement therapy, health-related quality of life, life after stroke, lower extremity, rehabilitation, SF-36, stroke, timing of therapy

## Abstract

**Introduction:**

The aim of this study was to explore potential effects of lower extremity Constraint-Induced Movement Therapy (LE-CIMT) on Health-Related Quality of Life (HRQoL) after stroke, and the possible influence of descriptive characteristics.

**Materials and methods:**

Twenty men and 10 women (median age 53 years, IQR 45–59) participated in a single-arm, intervention study at an outpatient physiotherapy clinic. Participants underwent intensive, task-specific LE-CIMT for 6 h/day over 10 consecutive weekdays, followed by a home-exercise program until a 3-month follow-up. HRQoL was assessed using the SF-36 questionnaire, and motor function was evaluated with part of the Fugl-Meyer Assessment, Berg Balance Scale, 10-Meter Walk Test, 6-Minute Walk Test, and Timed Up and Go. Assessments were performed pre-intervention and at 3-month follow-up.

**Results:**

The SF-36 response rate was 50%. HRQoL improved significantly in the Physical Functioning (median change 10, IQR 5–15, *p* < 0.001, ES 0.75) and Vitality domains median change 5 (IQR 0–20, *p* = 0.013, ES 0.46). A negative correlation was observed between time from stroke to LE-CIMT and improvement in Physical Functioning (*r*_S_ = −0.456, *p* = 0.013), but not the Vitality domain.

**Conclusion:**

Our findings suggest that LE-CIMT may be associated to improvements in HRQoL, particularly in the Physical Functioning and Vitality domains after stroke. Earlier initiation of intervention appears beneficial. These findings need to be validated in randomized controlled trials.

## 1 Introduction

Stroke is a leading cause of death and life-long disability worldwide, often resulting in hemiplegia and reducing the patient’s ability to perform daily activities (1–3). Health-related quality of life (HRQoL) is decreased after stroke with reports of up to 35% reduction compared to the general population (3–7). Evaluating HRQoL is therefore a key component in assessing the effectiveness of rehabilitation from a patient’s perspective.

Constraint-Induced Movement Therapy (CIMT) was originally designed for upper-extremity rehabilitation (8), where it has been demonstrated to be an effective intervention for improving motor function after stroke (9–12), and is recommended in national clinical guidelines (13, 14). A corresponding protocol has since been adapted for lower extremity rehabilitation (15), retaining the main principles of upper extremity CIMT; encouraging use of the hemiparetic limb; high-intensity, task-specific, and supervised training; shaping techniques in which complex movements are broken down into manageable components and progressively integrated; and the use of a transfer package to facilitate the application of newly acquired skills in everyday life.

Motor function is a primary target of post-stroke rehabilitation and particularly walking endurance and balance have been identified as important contributors to influence HRQoL (16–18). We, along with others, have previously reported that Lower Extremity-CIMT (LE-CIMT) is an effective method for improving lower extremity motor function after stroke, with demonstrated benefits in walking ability, balance and functional skills (19–23). In the recently published European Stroke Organization guidelines on motor function rehabilitation after stroke, although LE-CIMT is not mentioned, one of its core components - high-intensity task-specific supervised training - is recommended for lower extremity rehabilitation (24). However, evidence regarding the effects of LE-CIMT on HRQoL remains very limited. A recent systematic review examining the effects of LE-CIMT on motor function suggests that individuals treated with LE-CIMT may experience higher HRQoL than those treated with conventional physiotherapy, though this conclusion rests on evidence from only three studies (22).

The aim of this study was to investigate the effect of LE-CIMT on HRQoL, as measured by the SF-36 questionnaire, among individuals with hemiparetic stroke in an outpatient clinical setting. A secondary aim was to examine whether any observed effects were associated with descriptive characteristics.

## 2 Materials and methods

### 2.1 Study design

This single-arm, intervention study was conducted between the years 2003 and 2018, at an outpatient clinic in Stockholm, Sweden, specializing in upper- and lower extremity CIMT (19, 20, 25).

Clinical data, including outcome measures and descriptive characteristics, for patients who received LE-CIMT were collected retrospectively for the years 2003–2014 and thereafter prospectively to 2018. No changes were made in the study protocol, assessments or patient selection from the prospective to the retrospective part of the study.

Assessments were conducted in a standardized manner according to a written protocol, by the physiotherapist who delivered the intervention (second author). To minimize potential bias, results from previous assessments were not available to the physiotherapist at the time of re-assessment.

Written informed consent was obtained from all participants prior to study inclusion. Ethical approval was obtained from the regional Board of Research Ethics in Umeå, Sweden (now the Swedish Ethical Review Authority) (Dnr. 2013–327-31 M). The study was conducted in accordance with the Declaration of Helsinki.

Patients received LE-CIMT for 6 h/day over 10 consecutive weekdays and were assessed using physical performance tests at three time points: pre-LE-CIMT (baseline), post-LE-CIMT (data not included in the present study), and at a 3-month follow-up. Physical outcomes for a larger cohort have been reported previously (19, 20). The SF-36 questionnaire was administered only at baseline and at the 3-month follow-up because it assesses HRQoL over the preceding 4 weeks, and a post-LE-CIMT assessment would therefore have resulted in substantial overlap with the baseline assessment and intervention period.

### 2.2 Participants

The study cohort represents a subset of a larger cohort previously described with respect to physical outcomes in Marklund et al. and Sefastsson et al. (19, 20).

Patients were referred from local hospitals for LE-CIMT, which was provided as part of routine clinical care following inpatient rehabilitation after acquired brain injury. Patients with unstable medical conditions, as assessed by the physician, were not referred.

The outpatient clinic offered LE-CIMT to patients who demonstrated sufficient motivation to participate and were able to understand verbal and/or written instructions and walk indoors without a walking aid. Training started no earlier than 1 month post brain injury.

**Figure 1:**
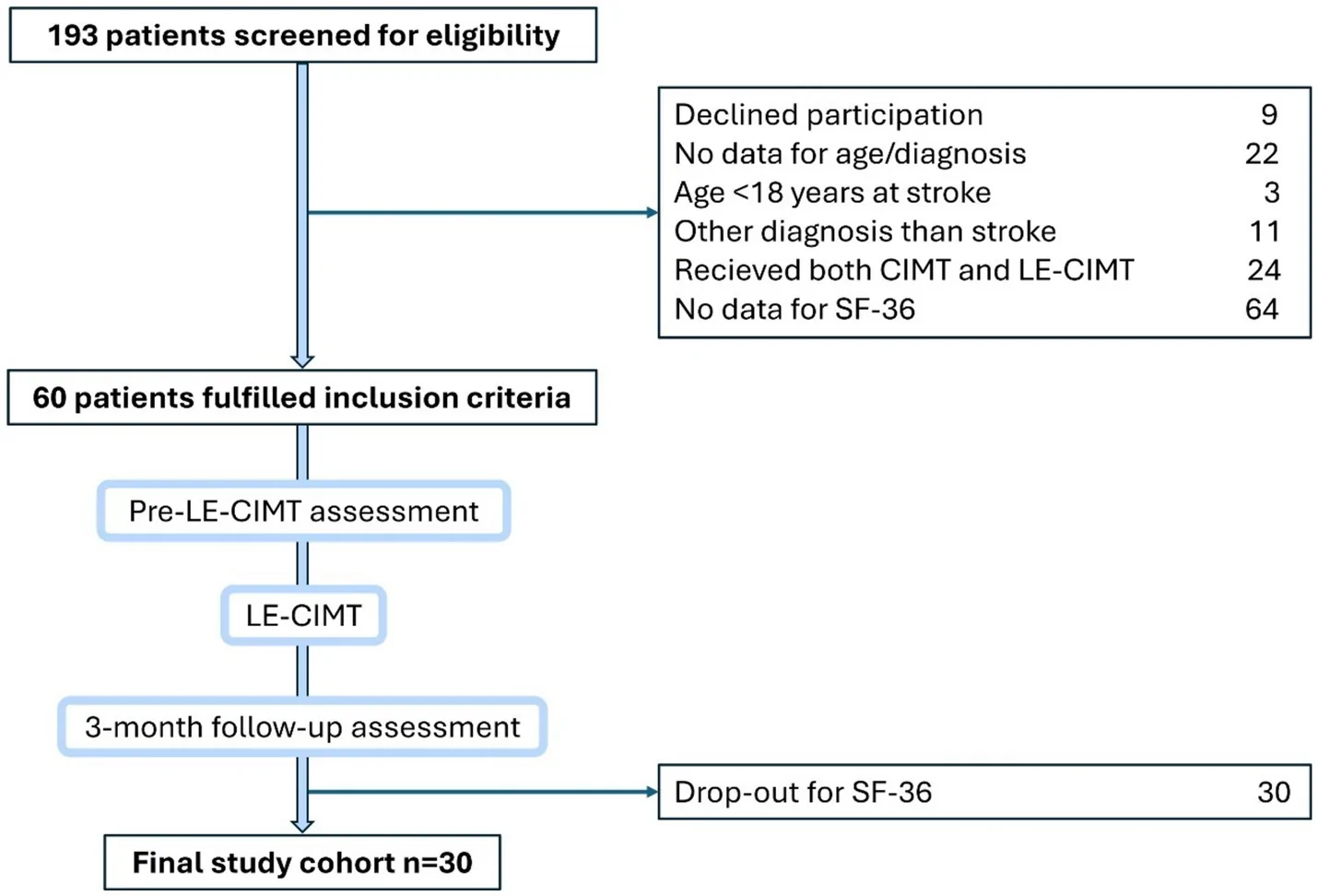
Flow-chart showing study design and number of excluded patients. LE-CIMT—Lower-Extremity Constraint-Induced Movement Therapy; SF-36—Short Form 36 survey.

Patients who had received, or were going to receive their first-ever LE-CIMT were invited to participate in the study if they met the following inclusion criteria: (1) first ever stroke, (2) age >18 years at time of stroke, (3) provision of written informed consent, (4) valid data for LE-SF-36. Missing HRQoL data at either the pre-LE-CIMT or 3-month follow-up assessment was an exclusion criterion in the final study cohort (Figure 1).

### 2.3 Intervention

Participants received individually tailored LE-CIMT in groups of 3–4, led by an experienced physiotherapist (the second author AS), as previously described in detail (19, 20). The training focused on exercises aimed at maximizing use of the hemiparetic leg and adhered to the core principles of the LE-CIMT protocol with some modifications (15):

(1) Intensive, task-specific, massed practice, 6 h per day over 10 consecutive weekdays—Each day consisted of seven 45 min sessions focusing on balance and strength, coordination and mobility targeting the leg, ankle and foot, as well as indoor and outdoor gait, functional training and passive muscle stretching. Example exercises included picking up objects from the ground, sit-to-stand transfers, and turning on different surfaces, walking, running, treadmill and stair training, unilateral cycling and leg press using the weaker leg. Training programs were revised daily to ensure that exercises remained appropriately challenging.
(2) Motor training based on shaping strategy—Complex motor tasks were practiced stepwise by gradually increasing the level of difficulty and eventually combining the parts into complete functional movements (26). This approach requires thorough assessment of the patients’ motor deficits. Frequent verbal feedback was provided by the physiotherapist to encourage and improve performance.
(3) Encouraging use of the hemiparetic leg—throughout the intervention period, patients were strongly encouraged to use the weaker leg. General oral and written instructions were provided to increase weight-bearing on the weaker leg during standing, stand-to-sit and sit-to-stand transitions, stair climbing, and during active leg lifting. Individualized home exercises were also provided for the two weekends included in the intervention period.

During part of gait training, a constraining splint (Knee Immo Art. No 47500, Camp Scandinavia AB, Helsingborg, Sweden) was used to maintain the stronger leg in an extended position, thereby promoting increased weight-bearing and use of the weaker leg. The extent of splint use was determined by the physiotherapist based on the type of activity and the patient’s functional response. The use of a constraining device in LE-CIMT is debated (15) as this may negatively influence gait patterns. However, a systematic review reported that incorporating a constraint in LE-CIMT led to greater improvement in motor function compared to LE-CIMT without constraint (22).

(4) Transfer package—the patients received individualized home training programs to be used in the period between LE-CIMT and the 3-month follow-up. Each program was developed in collaboration with the patient to determine appropriate training exercises and frequency. Patients were instructed to keep a diary of their home training. Adherence to the program was discussed at the 3-month follow-up, although not systematically recorded.

### 2.4 Outcome measures

#### 2.4.1 SF-36

HRQoL was assessed using the Swedish standard version of the Short Form-36 Health Survey (SF-36). The SF-36 questionnaire is a generic measure of self-perceived HRQoL that has been validated in stroke patients (4, 27–29), and consists of 36 items of which 35 assess eight aspects of HRQoL - Physical Functioning, Physical Role, Bodily Pain, General Health, Vitality, Social Functioning, Emotional Role and Mental Health - evaluating the preceding 4 weeks. The last question of the SF-36 is not included in the eight domains and pertains to perceived health during the last year. At the initiation of the study in 2003, the SF-36 questionnaire was validated for use in a Swedish stroke population (4), and was well established in both research and clinical practice in Sweden. Although stroke-specific instruments became more widely used during the study period, SF-36 was retained throughout the study period to ensure consistency in data collection and comparability of outcomes over time.

Results were analyzed for the individual domains as well as for the physical and mental component summaries (PCS, MCS). Each domain comprises 2–10 questions and was transformed into domain scores according to the Swedish Manual for SF-36 (30). Domain scores range from 0 to 100 where 100 is full perceived quality of life. Minimal Clinically Important Difference (MCID) for the Vitality domain has been reported to range from 5.58 to 15.43 (31). A literature search did not identify any established MCID values for the remaining SF-36 domains.

Component summary scores were calculated according to the SF-36 manual for summary scales using principal component analysis (32). Both PCS and MCS include all eight domains but are weighted differently with Physical Functioning, Physical Role, Bodily Pain and General Health mainly contributing to PCS and Vitality, Social Functioning, Emotional Role and Mental Health mainly contributing to MCS. Scoring involves three steps: (1) standardization of scales into z-scores, (2) aggregation of weighted scale scores, (3) transformation of summary scores into norm-based T-scores. MCID for PCS has been reported to be between 1.8 and 3.0, with 2.5 units recommended for clinical trials (33).

The SF-36 questionnaire was administered and completed at pre-LE-CIMT for baseline assessment. A second questionnaire was provided at the post-LE-CIMT visit together with both oral and written instructions for participants to complete it at home prior to the 3-month follow-up and return it at their scheduled clinic visit. Information was given that there would be no individual feedback on the results of SF-36.

If a participant forgot to bring the completed questionnaire to the clinic, they were offered the opportunity to complete it on site. Alternatively, a new questionnaire along with a pre-paid, pre-addressed envelope was provided for home completion and return by mail. No data were collected regarding reasons for non-completion of the SF-36 questionnaire.

#### 2.4.2 Physical outcomes

Assessments of physical outcomes have previously been described in detail (19, 20).

#### 2.4.3 Walking ability

Walking speed was assessed with the 10-Meter Walk Test at self-selected speed (10MWT) (34). Performance was recorded in seconds and results presented as m/s. MDC for 10MWT at self-selected speed has been calculated to be 0.04 m/s for individuals managing household ambulation, 0.12 m/s for limited community ambulation and 0.19 m/s for full community ambulation (35). It is estimated that full community ambulation demands a walking speed of >0.8 m/s (36) and approximately 1.3 m/s is commonly considered normal walking speed for healthy adults under 80 years of age (37).

Walking endurance was assessed using the 6-Minute Walk Test (6MWT) (38) where the patient walked continuously back and forth a distance of 10 m, for 6 min. Participants were instructed to walk as far as possible and were informed of the remaining time at 3 and 5 min. No verbal encouragement was given during the 6 min. Results were recorded in meters. The MDC has been reported to range from 28 to 52 meters (39).

#### 2.4.4 Motor function, functional mobility and balance

Lower extremity motor function was assessed with Fugl-Meyer Assessment for lower extremity, subscale E (40) without the tests for reflex (hereafter FMA). Maximum score was 22 points.

Functional mobility and balance were assessed with the Timed Up and Go (TUG) test (41) where the participant begins seated in a chair with armrests, stands up, walks 3 meters at maximum speed, turns around, walks back, and sits down. The TUG test has been shown to be valid and reliable and is commonly used to evaluate stroke patients (42). MDC for the TUG test is 8 s (43) or 3.2 s in chronic stroke patients (44).

Functional balance was assessed with the Berg Balance Scale (BBS). The BBS rates the patient’s ability to maintain balance while performing a functional task. It consists of 14 items on a five-point scale (0–4, maximum score 56 points) (45). The BBS has been found valid and reliable in the stroke population with a MCD of 2.7 points for chronic stroke (44).

### 2.5 Data presentation and statistical analysis

Descriptive statistics for baseline characteristics, including FMA, BBS and 10MWT at self-selected speed, were summarized for both the study cohort and the non-responders to SF-36. Exact age at stroke was known for all participants except one; for this individual, the age was imputed by subtracting the median time from stroke to CI from the age at LE-CIMT. Drop-out analyses were performed comparing baseline characteristics between the study cohort and drop-out for SF-36. Data are reported as frequencies and percentages for nominal variables, and as medians and interquartile ranges (IQR) for continuous variables. Distribution of continuous variables was assessed using histograms, Q-Q plots, and Shapiro–Wilk test. As the data included were mainly non-normally distributed variables and due to relatively small sample size, non-parametric tests were applied throughout the statistical analysis. No imputation was performed for missing data. Instead, analyses were conducted using complete-case data for each outcome.

Between-group comparisons were conducted using the Chi-square or Mann–Whitney U-tests, as appropriate. Within-group comparisons were performed using Wilcoxon matched-pairs signed-ranks test. A two-tailed *p*-value of <0.05 was considered statistically significant.

Effect size (ES) for Wilcoxon signed-rank test was calculated as r = Z/(√ n), where Z is the Z-statistics from the Wilcoxon signed-rank test and n is the total number of observations (46). ES (r) were interpreted according to Cohen’s criteria where │r│ = 0.1–0.3 indicates a small effect, │r│ = 0.3–0.5 a medium effect, and │r│ > 0.5 a large effect (47, 48).

Correlations between changes in HRQoL and descriptive variables were analyzed using Spearman’s rank correlation (19, 20). Power calculations showed that, with a sample size of 30, the study had 80% power to detect Spearman correlation coefficients of approximately 0.52. Therefore, weaker associations may have remained undetected, and non-significant findings should be interpreted with caution.

Three extreme outliers (>3 × IQR) for time from stroke onset to LE-CIMT (time to LE-CIMT) were identified using boxplots in SPSS. As these represented valid observations, they were retained in the primary analyses. Additional sensitivity analyses excluding the identified extreme outliers were performed.

Analyses were performed using the IBM SPSS Statistics software, version 29.0.1.0. (IBM Corp., Armonk, NY, United States). Figures were generated using the GraphPad Prism 10, version 10.3.0 (San Diego, CA, United States).

## 3 Results

### 3.1 Inclusion and response rate

Of the 193 patients who received LE-CIMT at the outpatient clinic and were screened for eligibility, 60 met the inclusion criteria. However, due to a 50% response rate for SF-36 questionnaire, the final study cohort consisted of 30 participants (Figure 1).

### 3.2 Descriptive characteristics and physical outcomes

Descriptive characteristics are presented in Table 1. Participants in the current study had a median age of 53 years (range 31–64). Baseline motor function was comparatively high, with a median self-selected walking speed of 0.9 m/s and a median BBS score of 54 out of 56 points. Time from stroke onset to intervention varied from 3 to 102 months (median 12 months, IQR 8–17). Thus, most participants were in the chronic post-stroke phase, with only 20.7% initiating LE-CIMT within 6 months of stroke onset. Three participants had extreme values with regard to time to LE-CIMT (54, 99 and102 months).

**Table 1:** Baseline descriptive characteristics and drop-out analysis.

Baseline characteristics	Study cohort (*n* = 30)		Drop-out for SF-36 (*n* = 30)		Study cohort vs. drop-out
	*n* (%)	Median (IQR)	*n* (%)	Median (IQR)	*p*-value
Gender (F/M)	10/20 (33.3/66.7)		13/17 (43.3/56.7)		0.426
Age at stroke (years)		53 (45–59)		49 (40–58)	0.214
Time from stroke to LE-CIMT (months)		12 (8–17) ^1^		9 (5–15)	0.093
Type of stroke (infarction/haemorrhagic/not specified)	18/9/3 (60.0/30.0/10.0)		18/9/3 (60.0/30.0/10.0)		>0.999
Affected side (right/left)	17/12^1^ (58.6/41.4)		19/11 (63.3/36.7)		0.711
FMA, pre-LE-CIMT (points)		16 (12–17)		17 (13–21) ^3^	0.282
Timed up and go, pre-LE-CIMT (s)		15 (12–22) ^4^		14 (10–19) ^5^	0.433
BBS, pre-LE-CIMT (points)		54 (50–56) ^1^		51 (49–56) ^2^	0.675
10 m walk test, self-selected speed, pre-LE-CIMT (m/s)		0.9 (0.6–1.0)		0.8 (0.5–1.0) ^3^	0.395
6-min walk test, pre-LE-CIMT (m)		284 (224–396) ^2^		307 (240–373) ^5^	0.838

^x^Number of missing data points in superscript. Descriptive statistics for final study cohort and drop-out for SF-36 at 3 months follow up, including p-values for the drop-out analysis. Between-group comparisons were performed using Mann–Whitney U test or Chi-square test, as appropriate. Number of missing data points in superscript. F, female; M, male; LE-CIMT, Lower-Extremity Constraint Induced Movement Therapy; FMA, Fugl-Meyer Assessment subscale E without reflex tests; BBS, Berg Balance Scale; SF-36, Short Form 36 survey; n, number of participants.

The present study cohort showed significant improvements in motor function, from pre-LE-CIMT to follow-up, including FMA (median difference 1.5 points, IQR 0.0–3.0); BBS (median difference 1.0 points, 0.0–4.0); 10mWT (median difference self-selected speed 0.1 m/s, 0.1–0.3); 6MWT (median difference 70 m, 37–107); and TUG (median difference −3 s, −7 to −1), all *p* < 0.001. Hence, 6MWT exceeded the MDC of 28–52 m, ES 0.83.

### 3.3 Dropout and sensitivity analyses

Dropout analysis comparing participants included in the final study cohort with those who did not complete the SF-36 questionnaire showed no significant differences in baseline descriptive characteristics or measures of motor function (Table 1).

As a sensitivity analysis, all analyses of changes in HRQoL from baseline to the 3-month follow-up and all correlation analyses and were repeated after excluding the three participants with extreme values for time to LE-CIMT. The results were largely unchanged, except that the correlations between change in the Physical Functioning domain score and time to LE-CIMT, and between change in MCS score and change in BBS score, were no longer statistically significant. Neither association reached the predefined threshold for adequate statistical power. As the extreme values represented valid observations, the primary analyses are presented for the complete study cohort.

### 3.4 SF-36 domains and component summaries

**Figure 2:**
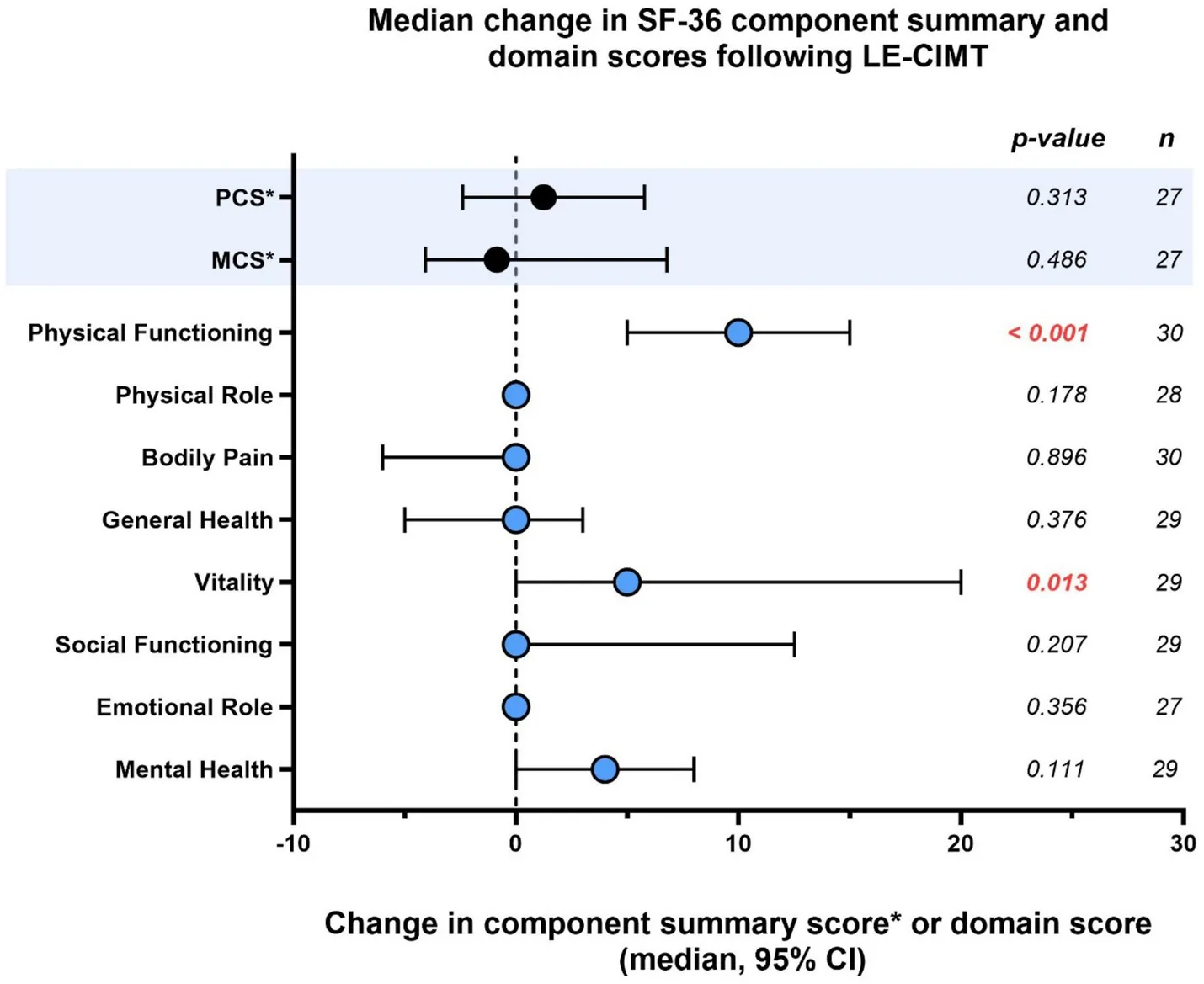
Median change and 95% CI in SF-36 domain scores from baseline (pre-LE-CIMT) to the 3-month follow-up. The SF-36 domains physical functioning and vitality showed statistically significant improvements. Within-group comparisons were performed using Wilcoxon signed ranks test. LE-CIMT—lower-extremity constraint-induced movement therapy, HRQoL—health-related quality of life, IQR—interquartile range, SF-36—short form 36 survey, *n*—number of participants, 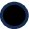 Component summary scores, 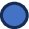 Domain scores. *SF-36 summary scores, significant *p*-values in red.

There were significant increases in HRQoL from pre-LE-CIMT to follow-up in both the Physical functioning domain (median change 10, IQR 5–15, *p* < 0.001, ES 0.75), and the Vitality domain (median change 5, IQR 0–20 *p* = 0.013, ES 0.46) (Figure 2 and Table 2). Improvement in the Vitality domain thus approached the MCID, reported to be between 5.58 and 15.43 (31).

**Table 2:** SF-36 results and correlations.

SF-36 summary score or domain	Median SF-36 score (IQR) *n* = 30		Correlation with time from stroke onset to LE-CIMT, *n* = 30	
	Baseline	3-month follow-up	R_S_	*p*-value
PCS	37.81 (32.06–44.58)^2^	43.31 (36.65–46.78)^1^	- 0.555^4^	**0.003**
MCS	58.66 (47.31–62.74)^2^	61.15 (51.75–63.45)^1^	0.273^4^	0.178
Physical functioning domain	44 (30–65)	62.5 (45–80)	−0.456^1^	**0.013**
Physical role domain	0 (0–37.5)^2^	25 (0–50)	−0.538^3^	**0.004**
Bodily pain domain	84 (62–100)	84 (64–100)	−0.553^1^	**0.002**
General health domain	82 (67–92)^1^	79.5 (62–92)	0.034^2^	0.862
Vitality domain	65 (50–75)^1^	72.5 (60–80)	−0.180^2^	0.360
Social functioning domain	75 (62.5–100)^1^	87.5 (62.5–100)	0.200^2^	0.308
Emotional role domain	100 (33.3–100)^2^	100 (66.7–100)^1^	−0.085^4^	0.679
Mental health domain	80 (70–92)^1^	88 (76–92)^1^	0.080^2^	0.685

*Number of missing data points in superscript. SF-36 summary and domain scores and IQR for baseline (pre-CIMT) and 3-month follow-up. Also shown are correlation coefficients and *p*-values for Spearman correlation with time from stroke onset to LE-CIMT. PCS, Physical component summary; MCS, Mental component summary; LE-CIMT, Lower Extremity Constraint-Induced Movement Therapy; n, number of participants.

No significant differences were observed in the physical or mental component summary scores (median difference pre-LE-CIMT to follow-up: PCS 1.24, IQR −3.66 to 6.51, *p* = 0.313; MCS −0.87, IQR −4.25 to 7.29, *p* = 0.486) (Figure 2 and Table 2).

### 3.5 Correlations

Age, gender, or affected side were not correlated to change in HRQoL from baseline to the 3-month follow-up in the present cohort. A shorter time to LE-CIMT was significantly correlated to increased scores in PCS and the SF-36 domains Bodily pain, Physical Role and Physical Functioning (Table 2).

In addition, statistically significant correlations were observed between change in Bodily Pain score and change in 6MWT distance (*r*_S_ = 0.404, *p* = 0.045, *n* = 25), change in MCS score and change in BBS score (*r*_S_ = −0.406, *p* = 0.040, *n* = 26), and change in Social functioning score and change in TUG time (*r*_S_ = −0.511, *p* = 0.009, *n* = 25). No other significant correlations were observed between changes in HRQoL and changes in physical outcomes.

## 4 Discussion

This single-arm, uncontrolled intervention study investigated the potential effects of 60-h LE-CIMT delivered in a real-world outpatient rehabilitation setting, on self-perceived HRQoL in 30 middle-aged individuals, most of whom were in the chronic phase after stroke. Compared with baseline, participants had significantly higher SF-36 scores in the Physical Functioning and Vitality domains at the 3-month follow-up. A shorter time from stroke onset to intervention was also associated with greater increases in the physical aspects of HRQoL.

The increase in the Physical Functioning domain score showed a large effect size. While the observed median improvement of 10 points is of similar magnitude to the MCID reported for the Vitality domain (31), no MCID has been established for the Physical Functioning domain. Consequently, the clinical relevance of the observed change remains uncertain. Although comparisons between studies should be interpreted with caution as different instruments were used to assess quality of life, our findings are largely consistent with those of a randomized controlled trial reporting improvements in the total score for Stroke-Specific Quality of Life (SSQoL) and the mobility subdomain (49). In contrast, another study using the SSQoL did not demonstrate statistically significant improvements in HRQoL after LE-CIMT (50). These discrepancies may be related to differences in study design, limited statistical power due to the small sample size, or differences in training dose, as participants in latter study received a total of 15 h of training (50), compared with 60 h in the present study.

As previously reported in a larger cohort from the same clinical setting (19, 20), the present subset also demonstrated improvements in objective measures of motor function, including balance, walking ability and functional mobility following LE-CIMT. Although the present study had limited statistical power to detect associations between changes in physical function and HRQoL, improvements in physical function may have contributed to the observed increases in the physical aspects of self-perceived HRQoL. This is consistent with previous studies reporting a positive association between HRQoL assessed with the SSQoL and BBS scores (51) and a recent systematic review and meta-analysis suggesting that LE-CIMT may improve physical aspects of quality of life after stroke (22).

The observed improvement in the SF-36 Vitality domain approached the MCID. This may reflect that participants experienced increased energy levels and reduced fatigue in their daily life following high-intensity LE-CIMT. However, vitality and fatigue are likely to be influenced by factors beyond physical training alone. Previous studies have suggested that CIMT may facilitate social interactions among participants, opportunities to share experiences with peers, and support from the physiotherapist (52, 53), all of which may have contributed to non-specific treatment effects and partly explain the observed improvement in the Vitality domain. Although physical therapy is often considered beneficial for fatigue management, a recent systematic review found limited evidence supporting its effectiveness in individuals with stroke (54). Nevertheless, the present findings are consistent with the possibility that intensive LE-CIMT may be associated with improvements in vitality and fatigue-related aspects of HRQoL.

A shorter time from stroke onset to intervention was associated with higher scores in physical aspects of HRQoL including PCS. Given the uncontrolled intervention design, causal inference is limited, and the observed associations should therefore be interpreted with caution. Nevertheless, the findings suggest that earlier initiation of LE-CIMT may be associated with greater improvements in self-perceived physical aspects of HRQoL after stroke. This is consistent with the well-established benefits of early post-stroke rehabilitation (14, 55), which have been linked to a greater capacity for activity-driven neuroplasticity during the earlier stages of recovery, although the potential for rehabilitation persists beyond the acute and subacute phases (56). The wide range in time from stroke onset to LE-CIMT in the present study may therefore have contributed to an underestimation of the potential benefits associated with earlier intervention.

### 4.1 Strengths and limitations

A strength of this study is that LE-CIMT was delivered in a real-world outpatient clinical setting, providing insights into outcomes achievable in routine clinical practice. Our findings suggest that high-intensity LE-CIMT may be a feasible rehabilitation intervention for individuals with stroke in the subacute and chronic stages. However, these results should be interpreted with caution given the study design. The absence of a control group limits causal inference and increases the risk that the observed changes were influenced by factors other than LE-CIMT, including the non-specific treatment effects discussed above. In addition, the small sample size limited statistical power, resulting in less precise estimates and reducing the ability to detect potentially meaningful differences and associations. Although SF-36 has been validated for stroke patients (4), the use of a generic rather than a stroke-specific HRQoL instrument limits comparability with more recent studies and may have reduced sensitivity to stroke-specific changes in HRQoL.

Another limitation of this study is that data were collected over a prolonged period of 16 years, retrospectively for the first part and prospectively thereafter. Although the study protocol, inclusion procedures and outcome measures remained unchanged, retrospective data collection may have limited the ability to monitor data collection procedures and ensure complete documentation. While each participant served as their own control, temporal changes during the study period, including changes in referral patterns, rehabilitation practices outside the LE-CIMT program, and the use of assistive devices and medical treatments, may still have influenced the observed outcomes independently of the intervention. In addition, adherence to the home exercise program between intervention and follow-up was not monitored, and spontaneous recovery and regression to the mean cannot be excluded, although both are expected to be limited in individuals in the chronic phase after stroke.

The study is also subject to potential sources of bias. First, the 50% response rate for the SF-36 raises concerns about potential attrition bias. Although dropout analysis showed no differences in baseline characteristics or motor function between the final study cohort and participants which did not complete the SF-36 questionnaire, the analysis may have been underpowered to detect smaller between-group differences. Furthermore, information was not collected regarding the reasons for non-completion, precluding further analysis. Second, all physical outcome assessments were performed by the same physical therapist who delivered the intervention, introducing a potential risk of detection bias. This was partly mitigated by ensuring that previous assessment results were not available to the assessor at the time of reassessment. Conversely, the use of a single assessor may have improved consistency and reduced potential inter-rater variability. Finally, the self-reported nature of the SF-36 introduces the risk of response bias, as responses may have been influenced by expectations or social desirability.

Notably, the present study cohort primarily consisted of middle-aged stroke survivors, none older than 64 years. While this limits generalizability to older stroke populations, the findings provide important information for a group in which HRQoL has been reported to be particularly impaired (4). Furthermore, as many middle-aged stroke survivors remain of working age, rehabilitation interventions such as LE-CIMT that may improve motor functioning and quality of life could have important personal and socioeconomic implications.

## 5 Conclusion

The present findings suggest that LE-CIMT may be associated with improvements in self-perceived HRQoL, particularly in the Physical Functioning and Vitality domains, among middle-aged individuals with relatively good baseline motor function after stroke. These findings highlight the importance of including HRQoL as an outcome in future evaluations of LE-CIMT In addition, the results indicate that earlier initiation of LE-CIMT may be associated with greater improvements in physical aspects of self-perceived HRQoL. However, given the single-arm, uncontrolled study design these findings should be interpreted with caution, and further research, including randomized controlled trials with larger sample sizes and longer follow-up, is needed to confirm these findings.

## Data Availability

The raw data supporting the conclusions of this article will be made available by the authors, without undue reservation.
